# *GREM1* is expressed in the cancer-associated myofibroblasts of basal cell carcinomas

**DOI:** 10.1371/journal.pone.0174565

**Published:** 2017-03-27

**Authors:** Hye Sung Kim, Myung Soo Shin, Min Seok Cheon, Jae Wang Kim, Cheol Lee, Woo Ho Kim, Young Sill Kim, Bo Gun Jang

**Affiliations:** 1 Department of Pathology, Jeju National University School of Medicine and Jeju National University Hospital, Jeju, South Korea; 2 Department of Plastic Surgery, Jeju National University School of Medicine and Jeju National University Hospital, Jeju, South Korea; 3 Department of Dermatology, Jeju National University School of Medicine and Jeju National University Hospital, Jeju, South Korea; 4 Department of Pathology, Seoul National University College of Medicine, Seoul, South Korea; King Faisal Specialist Hospital and Research Center, SAUDI ARABIA

## Abstract

Cancer-associated fibroblasts (CAFs) play important roles in cancer progression through their complex interactions with cancer cells. The secreted bone morphogenetic protein antagonist, gremlin1 (GREM1) is expressed by the CAFs of basal cell carcinomas (BCCs), and promotes the growth of cancer cells. In this study, we investigated the expression of *GREM1* mRNAs in various benign and malignant skin tumors, including various BCC subtypes. Analysis by RNA in situ hybridization (ISH) revealed that fibroblasts in the scar tissue expressed *GREM1* and α-smooth muscle actin (α-SMA), whereas resident fibroblasts in the dermis of the normal skin did not express *GREM1*. Real-time polymerase chain reaction analysis showed significantly higher *GREM1* expression in skin cancers and pilomatricomas (PMCs) than in other benign skin tumors. Tissue microarrays analyzed by RNA ISH for *GREM1* expression also demonstrated that 23% of BCCs, 42% of squamous cell carcinomas, 20% of melanomas, and 90% of PMCs were positive for *GREM1* expression, whereas trichoepitheliomas, eccrine poromas, hidradenomas, and spiradenomas were negative for *GREM1* expression. Most BCCs that were *GREM1* expression positive were of desmoplastic or mixed subtypes, and *GREM1* expression was localized to activated myofibroblasts at the tumoral-stromal interface. Interestingly, most PMCs harbored *GREM1*-expressing fibroblasts, probably because of the inflammatory responses caused by foreign body reactions to keratin. Additionally, in BCCs, stromal *GREM1* expression had a strong correlation with CD10 expression. In conclusion, *GREM1* is frequently expressed by myofibroblasts in scars or in the stroma of basal cell carcinomas, suggesting that *GREM1* expression can be a marker for activated myofibroblasts in the cancer stroma or in scar tissue.

## Introduction

Cancer-associated fibroblasts (CAFs) are essential components of cancer microenvironments, and play critical roles in the cancer progression; they tend to aggregate peritumorally and encircle cancer cells that are invading adjacent normal tissues [1]. CAFs can originate from multiple precursors such as resident fibroblasts, smooth muscle cells, endothelial cells, and bone marrow (BM)-derived mesenchymal stem cells [2, 3]. CAFs secrete a wide spectrum of soluble factors including growth factors, chemokines, and cytokines, and contribute to the growth, migrations, epithelial mesenchymal transitions (EMTs), and metastases of the cancer cells. Thus, molecular markers of CAFs, such as fibroblast activation protein (FAP), C-X-C motif chemokine ligand 12 (CXCL12), and hepatocyte growth factor (HGF) are emerging as selective therapeutic targets in the cancer stroma [4]. Only a few studies on skin cancers have examined the molecular markers in CAFs for developing novel therapeutic strategies [5, 6].

Basal cell carcinomas (BCCs) have a characteristic fibromyxoid stroma with CAFs present around the tumor cells. In 2006, CAFs of human BCCs were first shown to express high levels of gremlin1 (*GREM1)* expression which promote the proliferation of cultured BCC cells [7]. Moreover, these *GREM1*-expressing CAFs were also observed in many other carcinomas [7]. GREM1 is a secreted bone morphogenetic protein (BMP) antagonist and is known to be involved in renal development [8, 9] and in the pathogenesis of nephropathy [10, 11]. Intestinal cryptal myofibroblasts and smooth muscle cells also express *GREM1* at colon crypts, which contribute in the formation of the intestinal stem cell niche [12]. Interestingly, aberrant epithelial *GREM1* expression leads to the development of hereditary mixed polyposis syndrome (HMPS) and traditional serrated adenomas [13]. Some reports on colon cancers have highlighted the stromal expression of *GREM1* at the invasion front [14, 15].

BCCs are classified into several subtypes, based on distinct histological features, into nodular, micronodular, desmoplastic, mixed, and superficial subtypes. However, detailed patterns of *GREM1* expression in these BCC subtypes remain undefined. In addition, *GREM1* expression in the stromal cells of benign skin tumors and of other malignant tumors such as SCCs and MNs is unknown. In particular, trichoepitheliomas (TEs) that often confound a differential diagnosis of BCCs have characteristic peritumoral stromal cells; this raise the question of whether there are any differences in stromal *GREM1* expression between BCCs and TEs that can be used as potential diagnostic feature. For the detection of *GREM1* expression, RNA in situ hybridization (ISH) has enabled the specific visualization of *GREM1* mRNA in human formalin-fixed paraffin-embedded (FFPE) specimens [7, 12, 13]. Therefore, in this study, we have used RNA ISH to investigate the stromal expression of *GREM1* in various skin tumors, and compared the expression of *GREM1* between benign and malignant tumors, and between the distinct BCC subtypes.

## Materials and methods

### Patients

A total of 152 FFPE tissues (normal skin, n = 6; scar tissues, n = 10; skin tumors, n = 160) were obtained from the punch or the excisional biopsy specimens at the Jeju National University Hospital, Jeju and at the Seoul National University Hospital, Seoul, Korea, from 2011 to 2015. Skin tumors included basal cell carcinomas (BCCs, n = 81), squamous cell carcinomas (SCCs, n = 17), malignant melanomas (MNs, n = 5), pilomatricomas (PMCs, n = 10), trichoepitheliomas (TEs, n = 16), eccrine poromas (EPs, n = 11), spiradenomas (SPAs, n = 10), and hidradenomas (HDAs, n = 9). Clinicopathological characteristics of patients, including their sex, age, and tumor sizes are summarized in Table 1. All hematoxylin and eosin-stained slides were reviewed independently by two dermatopathologists (B.G.J and C.L.). The BCCs were classified into nodular, micronodular, superficial, desmoplastic, and mixed subtypes. Mixed subtype refers to BCCs containing both nodular and desmoplastic areas. Additionally, surgically excised skin tumors, including 18 BCCs, 9 SCCs, 8 MNs, 8 PMCs, 7 TEs, 9 EPs, 10 HDAs, and 8 SPAs, were collected for real-time polymerase chain reaction (PCR) analyses. All data were analyzed anonymously and this study was approved by the Institutional Review Boards of the Jeju National University Hospital (2016-06-005) and the Seoul National University Hospital (1607-048-774).

**Table 1:** Clinicopathological characteristics of skin tumors.

	BCC (n = 81)	TE (n = 16)	SCC (n = 17)	MN (n = 5)	PMC (n = 10)	EP (n = 11)	HDA (n = 9)	SPA (n = 10)
**Sex**								
Male (%)	29 (36)	3 (19)	7 (41)	4 (80)	2 (20)	6 (55)	2 (22)	5 (50)
Female (%)	52 (64)	13 (81)	10 (59)	1 (20)	8 (80)	5 (45)	7 (78)	5 (50)
**Age (years)**								
Mean (s.d.)	75 (13)	49 (19)	69 (15)	64 (15)	9.8 (4.3)	56 (20)	44 (21)	54 (11)
Range	24–94	6–71	33–93	49–83	3–18	28–82	2–68	36–69
**Tumor size (cm)**								
Mean (s.d.)	1.0 (0.8)	1.2 (1.3)	1.8 (1.4)	4.3 (2.1)	1.7 (0.5)	1.3 (0.5)	1.5 (0.6)	1.3 (0.7)
Range	0.2–5.3	0.5–4.0	0.4–6.0	1.8–7.0	1.0–2.5	0.8–2.0	0.8–2.2	0.4–2.7

Abbreviation: BCC, Basal cell carcinoma; TE, trichoepithelioma; SCC, squamous cell carcinoma; MN, malignant melanoma; PMC, pilomatricoma; EP, eccrine poroma; HDA, hidradenoma, SPA, spiradenoma, s.d., standard deviation

### RNA extraction and quantitative real-time PCR

Representative tumor areas were manually dissected from one to four FFPE tissue sections (4-μm thick) from each paraffin block; total RNA from these sections was extracted with an RNeasy FFPE Kit (Qiagen, Valencia, CA, USA) according to the instructions provided by the manufacturer but with a slight modification, as described previously [16]. cDNAs were synthesized from 1–2 μg of RNA using random hexamer primers and the GoScript reverse transcription system (Promega, Madison, Wisconsin, USA). Real-time PCR was performed with a StepOne Plus real-time PCR system (Applied Biosystems, Foster City, CA, USA) using the Premix Ex Taq (Takara Bio, Shiga, Japan) according to the recommendations provided by the manufacturer. The cycling conditions were as follows: initial denaturation for 20 s at 95°C, followed by 40 cycles of 95°C for 1 s and 60°C for 20 s. The following TaqMan gene expression assays were used: (*GREM1*), and Hs0275899_g1 (*GAPDH*). *GAPDH* served as the endogenous control.

### Tissue microarray construction

Ten tissue microarrays (TMAs) containing samples from 94 BCCs, 17 SCCs, 5 MNs, 12 PMCs, 18 TEs, 7 EPs, 5 SPAs, 8 HDAs, 6 normal skin tissues, and 10 scar tissues were constructed. Briefly stated, one representative tumor area (4 mm in diameter) was extracted from individual FFPE tumors (donor blocks) and arranged in new recipient paraffin blocks (tissue array block) using a trephine apparatus (SuperBioChips Laboratories, Seoul, Korea).

### RNA in situ hybridization

Using previously described protocols, RNA in situ hybridizations (ISH) for *GREM1* were performed on 10 TMAs with the RNAscope FFPE assay kit (Advanced Cell Diagnostics, Inc., Hayward, CA, USA) [17]. Briefly, 4-μm TMA sections were baked at 60°C for 1 hour. Next, each of these sections was subjected to protease digestion followed by hybridization with *GREM1* probes for 2 hours. An horseradish peroxidase (HRP)-based signal amplification system was hybridized to the probes before color development with 3,3′-diaminobenzidine tetrahydrochloride (DAB). The housekeeping gene, ubiquitin C (UBC), and the bacterial gene, DapB, served as positive and negative controls, respectively. Samples with UBC that were easily visible under a 10 × objective lens were considered adequate, as recommended by the manufacturer. Positive staining was indicated by the presence of brown punctate dots in the nucleus or cytoplasm or both. The expression of *GREM1* was quantified according to the recommended scoring guidelines as follows: score 0, no staining or less than one dot per cell; score 1: one to three dots per cell (visible at 20–40 × magnification); score 2, four to 10 dots per cell and no or very few dot clusters (visible at 20–40 ×); score 3, more than10 dots per cell and less than10% positive cells with dot clusters (visible at 20 ×); score 4, more than10 dots per cell and >10% positive cells with dot clusters (visible at 20 ×). When the ISH score of sample was more than 2, the sample was considered to be positive for *GREM1*.

### Immunohistochemistry

Immunohistochemistry was performed on 4μm thick TMA sections using a BOND-MAX automated immunostainer and a Bond Polymer Refine Detection kit (Leica Microsystems, Wetzlar, Germany), according to the instructions provided by the manufacturer. The primary antibodies used were anti-BCL2 (1:50; Dako, Carpenteria, CA, USA), anti-CD34 (1:300; Immunotech, Marseille, France), anti-CD10 (1:200; Novocatra, Newcastle, UK), and anti-α-smooth muscle actin (α-SMA) (1:1000; Neomarkers, Lab Vision Corporation, Fremont, CA, USA). Staining for BCL2 was considered as positive when over 10% of tumor cell nuclei were strongly positively stained for BCL2. Staining for CD34, CD10, and α-SMA were considered as positive when over 10% of stromal fibroblasts showed positive cytoplasmic staining.

### Statistical analysis

Statistical analyses were performed with Prism version 5.0 (GraphPad Software, Inc., San Diego, CA, USA). Between-group comparisons of real-time PCR data were tested using Student’s *t*-test. The associations between *GREM1* positivity and the immunohistochemical markers were tested by Pearson chi-square test. A *P*-value < 0.05 was considered statistically significant.

## Results

### RNA in situ hybridization for *GREM1* in normal skin and scar tissue

**Fig 1:**
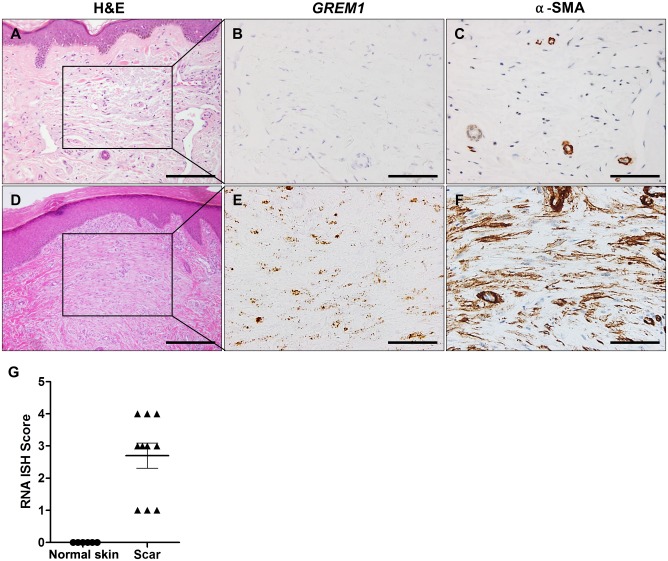
*GREM1* expression in normal skin and scar tissue. RNA in situ hybridization (ISH) for *GREM1* and immunohistochemical analysis for α-SMA was performed on the normal skin (n = 6) and scar tissues (n = 10). (A–C) Dermal fibroblasts of normal skin were negative for *GREM1* or α-smooth muscle actin (α-SMA). (D–F) Scar tissue fibroblasts were positive for both *GREM1* and α-SMA. (G) RNA ISH scores for *GREM1* in normal skin and scar tissues. Scale bar: 40 μm (A and D), 20 μm (B, C, E, and F).

Firstly, we validated the use of RNA ISH for detection of *GREM1* mRNAs in human FFPE specimens containing normal colon tissues, in which *GREM1*-expressing cells are known to be present. As previously shown, results of RNA ISH clearly demonstrated that the smooth muscle cells in the muscularis mucosa expressed *GREM1* (S1 Fig) [12]. Secondly, we examined *GREM1* expression in normal skin and scar tissues. Formation of scar is a non-tumorous condition in which fibroblasts proliferate and become activated. As expected, RNA ISH revealed that *GREM1* transcripts were not found in the resident dermal fibroblasts. On the other hand, a number of fibroblasts that had proliferated in the scar tissues highly expressed *GREM1* as well as α-SMA, demonstrating that *GREM1*-expressing myofibroblasts may also appear under physiological conditions such as wound healing (Fig 1).

### Real-time PCR analysis for *GREM1* in various skin tumors

**Fig 2:**
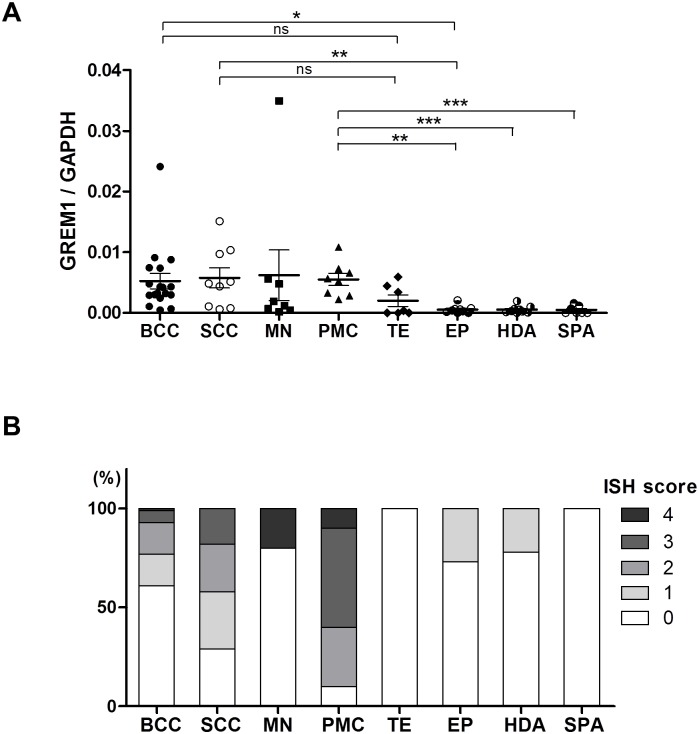
mRNA levels of *GREM1* in various skin tumors. (**A**) Real-time PCR analysis was performed to examine the expression of *GREM1* in a variety of skin tumors, including basal cell carcinomas (BCCs, n = 18), squamous cell carcinomas (SCCs, n = 9), melanomas (MNs, n = 8), pilomatricomas (PMCs, n = 8), trichoepitheliomas (TEs, n = 7), eccrine poromas (EPs, n = 9), hidradenomas (HADs, n = 9), and spiradenomas (SPAs, n = 7). *P < 0.05; **P < 0.00; 5***P < 0.001; ns, not significant. (**B**) The proportion of RNA in situ hybridization (ISH) scores for *GREM1* in skin tumors. Analysis of *GREM1* expression by RNA ISH was performed on tissue microarrays, including BCCs (n = 81), SCCs (n = 17), MNs (n = 5), PMCs (n = 10), TEs (n = 16), EPs (n = 11), HDAs (n = 9), and SPAs (n = 10). RNA ISH for *GREM1* was scored from 0 to 4, reflecting the intensity of *GREM1* expression.

Next, we assessed the transcription levels of *GREM1* in various benign and malignant skin tumors. The following FFPE samples were collected: BCCs (n = 18), SCCs (n = 9), MNs (n = 8), PMCs (n = 8), TEs (n = 7), EPs (n = 9), HDAs (n = 9), and SPAs (n = 7). Real-time PCR analysis showed that *GREM1* expression was higher in malignant tumors, such as BCCs (*P* < 0.05) and SCCs (*P* < 0.01) than in benign tumors, such as EPs, HDAs, and SPAs (Fig 2A). Although *GREM1* expression in melanomas was not significantly higher than in other tumors, one case had the highest level of *GREM1* among the tumors examined in this analysis. Notably, compared to other benign tumors, PMCs expressed remarkably higher levels of *GREM1*. In contrast, compared to other tumors, TEs showed no significant differences in *GREM1* expression.

### Stromal *GREM1* expression in benign and malignant skin tumors

**Fig 3:**
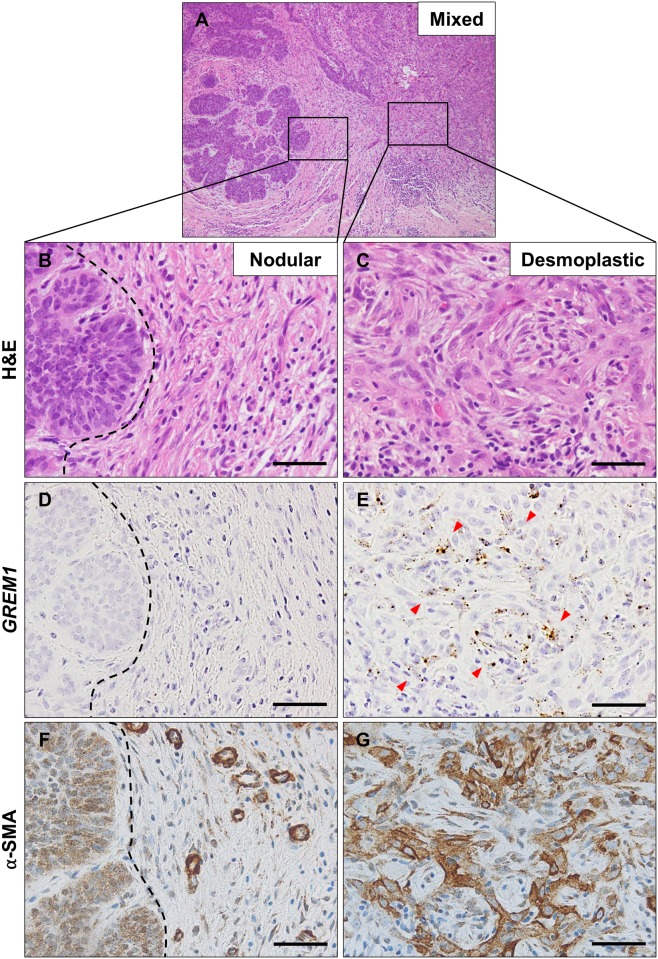
*GREM1* expression in mixed type basal cell carcinoma. A representative hematoxylin and eosin staining of a basal cell carcinoma (A) having both nodular (B) and desmoplastic (C) features. Fibroblasts around the nodular area (indicated by black dotted line) were negative for *GREM1* (D) and α-smooth muscle actin (F), whereas those around the desmoplastic area were positive for both *GREM1* (indicated by red arrow heads) (E) and α-SMA (G). RNA in situ hybridization for *GREM1* and immunohistochemical analysis for α-SMA. Scale bar: 25 μm.

**Fig 4:**
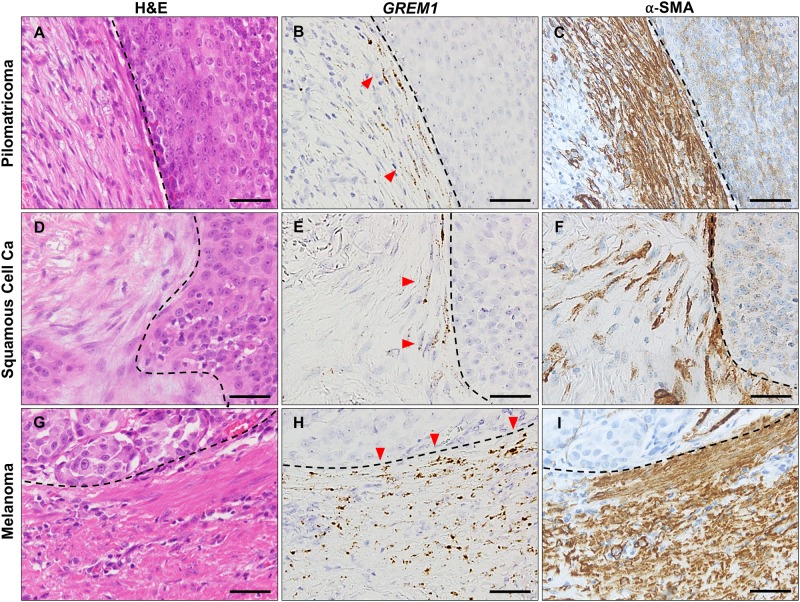
*GREM1* expression in the myofibroblasts at the tumoral-stromal interface. *GREM1*-positive myofibroblasts (indicated by red arrows) were frequently concentrated at the tumor-stromal interface (indicated by dotted line) in pilomatricoma (A–C), squamous cell carcinoma (D–F), and melanoma (G–I). RNA in situ hybridization for *GREM1* and immunohistochemical analysis for α-smooth muscle actin. Scale bar: 25 μm.

To specifically identify *GREM1*-expressing cells, we performed RNA ISH on tissue microarrays containing various samples of skin tumors. The results of RNA ISH are shown in Table 2 and Fig 2B. None of the epithelial components of skin tumors included in this study showed *GREM1* mRNA expression; *GREM1* mRNA localized to the stromal fibroblasts only. Of the malignant tumors, *GREM1* positivity (score >2) was observed in 19 cases (23%) of BCCs, in 7 cases (42%) of SCCs, and in one case (20%) of MN. Among benign skin tumors, only PMCs (9 cases, 90%) showed *GREM1* positivity. No *GREM1* positivity was observed in cases of TEs, EPs, HDAs, and SPAs. In BCCs, *GREM1* positivity was higher in the desmoplastic (43%) and mixed subtypes (39%) than in nodular (13%), micronodular (0%), and superficial subtypes (0%) (Table 3). In the cases of mixed BCC subtypes, only the myofibroblasts in the desmoplastic area expressed *GREM1* (Fig 3). In contrast, fibroblasts around nodular areas expressed neither *GREM1* nor α-SMA. Likewise, *GREM1*-expressing fibroblasts in PMCs, SCCs and MNs were observed in areas where activated myofibroblasts were present (Fig 4). In particular, fibroblasts immediately adjacent to the tumoral-stromal interface had high *GREM1* expression. Based on these findings, we suggest that *GREM1* expression is induced in myofibroblasts in the stroma, upon activation by invasive cancer cells or inflammation.

**Table 2:** RNA ISH scores for *GREM1* in various skin tumors.

	RNA ISH score	BCC (%)	SCC (%)	MN (%)	PMC (%)	TE (%)	EP (%)	HDA (%)	SPA (%)
***GREM1***	0	50 (62)	5 (29)	4 (80)	1 (10)	16 (100)	8 (73)	7 (78)	10 (100)
	1	12 (15)	5 (29)	0 (0)	0 (0)	0 (0)	3 (27)	2 (22)	0 (0)
	2	13 (16)	4 (24)	0 (0)	3 (30)	0 (0)	0 (0)	0 (0)	0 (0)
	3	5 (6)	3 (18)	0 (0)	5 (50)	0 (0)	0 (0)	0 (0)	0 (0)
	4	1 (1)	0 (0)	1 (20)	1 (10)	0 (0)	0 (0)	0 (0)	0 (0)
**Total**		81 (100)	17 (0)	5 (100)	10 (100)	16 (100)	11 (100)	9 (100)	10 (100)

Abbreviation: BCC, basal cell carcinoma; SCC, squamous cell carcinoma; MN, malignant melanoma; PMC, pilomatricoma; TE, trichoepithelioma; EP, eccrine poroma; HAD, hidradenoma; SPA, spiradenoma

**Table 3:** RNA ISH scores for *GREM1* in basal cell carcinomas subtypes.

	RNA ISH score	Nodular (%)	Micronodular (%)	Desmoplastic (%)	Mixed* (%)	Superficial (%)
***GREM1***	0	22 (73)	7 (100)	5 (36)	9 (39)	5 (83)
	1	4 (13)	0 (0)	4 (21)	5 (22)	1 (17)
	2	4 (13)	0 (0)	4 (29)	5 (22)	0 (0)
	3	0 (6)	0 (0)	1 (7)	4 (17)	0 (0)
	4	0 (1)	0 (0)	1 (7)	0 (0)	0 (0)
**Total**		30 (100)	7 (100)	14 (100)	23 (0)	6 (100)

* Mixed type refers to basal cell carcinomas showing both nodular and desmoplastic features.

### Associations between *GREM1* and the immunohistochemical markers in basal cell carcinomas

**Fig 5:**
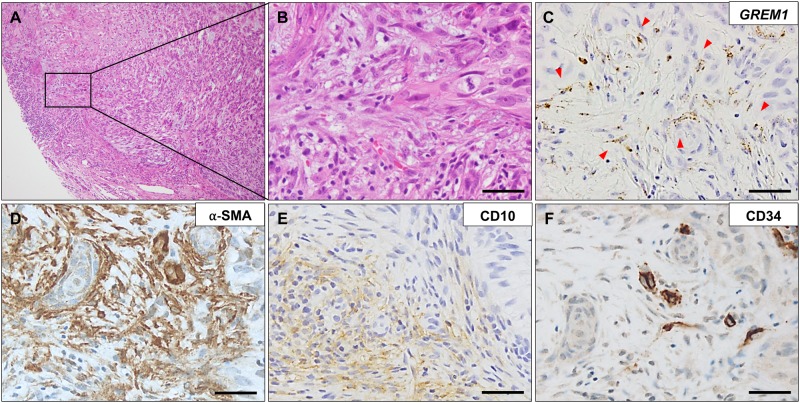
Association of *GREM1* expression with expression of stromal CD10 and CD34 in basal cell carcinoma. A representative picture of basal cell carcinoma with accompanying *GREM1*-positive myofibroblasts (indicated by red arrow heads) (A–D), in which stromal CD10 expression (E) was positive but that of CD34 (F) was negative. RNA in situ hybridization for *GREM1* and immunohistochemical analysis for α-smooth muscle actin, CD10, and CD34. Scale bar: 25 μm.

To enable the differential diagnosis of BCCs from TEs, several immunohistochemical markers have been studied, for example BCL2, CD10, and CD34, of which CD10 and CD34 are known to be expressed by peritumoral stromal cells [18]. To examine whether the stromal expression of *GREM1* has any correlation with the presence of these diagnostic markers, we performed immunohistochemical analysis for CD10, CD34, and BCL2. Expression of *GREM1* showed a strong association with stromal CD10 expression (Table 4). On the other hand, there was no association between expression of stromal *GREM1* and that of epithelial CD10. Expression of stromal CD34 or epithelial BCL2 did not have any correlation with *GREM1* expression. A representative case is shown in Fig 5.

**Table 4:** Associations between *GREM1* and immunohistochemical markers in basal cell carcinomas.

	*GREM1*		*P*-value^#^
	Negative (%)	Positive (%)	
**BCL2 (tumor)**			
Negative	20 (69)	9 (31)	0.314
Positive	38 (79)	10 (21)	
**CD34 (stroma)**			
Negative	35 (71)	14 (29)	0.179
Positive	27 (84)	5 (16)	
**CD10 (tumor)**			
Negative	27 (75)	9 (25)	0.812
Positive	34 (77)	10 (23)	
**CD10 (stroma)**			
Negative	49 (88)	7 (12)	0.000
Positive	12 (50)	12 (50)	
**α-SMA (stroma)**			
Negative	45 (94)	3 (6)	0.000
Positive	16 (50)	16 (50)	

^#^ Pearson Chi-Square test

## Discussion

In this study, we demonstrated that *GREM1* is frequently expressed by CAFs in the tumoral-stromal interface of invasive skin cancers, whereas the resident fibroblasts in normal skin do not express *GREM1*. Notably, we observed the appearance of *GREM1*-positive fibroblasts in scar tissue, showing that *GREM1* expression is not confined to cancer-associated stromal cells, but can also be a phenotypic marker for activated fibroblasts under physiological conditions. Cancers have been demonstrated to activate the latent wound-healing program in an exaggerated and prolonged manner [19]. Therefore, both CAFs and myofibroblasts in scar tissue may express *GREM1* possibly because of the activation of a shared molecular pathway. Considering that recently *GREM1* has been identified as a marker of mesenchymal stem cells in the bone marrow that can generate bone, cartilage, and the periepithelial intestinal mesenchymal sheath [20], it is also possible to hypothesize that *GREM1*-expressing myofibroblasts in the scar tissue and the cancer stroma originate from *GREM1*-positive mesenchymal stem cells in the bone marrow upon tissue destruction either by cancer invasion or inflammation.

Previously, Sneddon et al. have reported that most BCCs (80%, 12 of 15 cases) express *GREM1* [7]. In contrast, our results showed that only 28% (or up to 38% when including cases with score of 1) of BCCs (23 of 81 cases) were positive for *GREM1*. This discrepancy in the overall expression of *GREM1* can be explained in part by the differences between the two studies, *vis-à-vis* the use of RNA ISH and the criteria for *GREM1* positivity. In addition, our results suggest that the BCC subtypes included in the study can be important determinants of *GREM1* expression because desmoplastic or mixed BCC subtypes exhibited greater *GREM1*-positivity. Thus, if a study includes a larger number of desmoplastic or mixed subtypes, the *GREM1* positivity is expected to be higher. Histologically, desmoplastic and mixed subtypes are characterized by cancer cell invasion into the surrounding tissues, leading to stronger stromal reactions than those seen with other subtypes, and these subtypes have larger numbers of activated myofibroblasts around the cancer cells. On the other hand, CAFs in micronodular and superficial subtypes are relatively smaller in number, and are not in an activated state. These findings seem to indicate that stromal *GREM1* expression in BCCs largely depends on the extent of invasiveness of cancer cells and the damage sustained by adjacent stromal tissue.

As mentioned earlier, stromal *GREM1* expression has been demonstrated not only in BCCs but also in many invasive carcinomas, such as carcinomas of the esophagus, pancreas, colon, lung, breast, and mesotheliomas [7, 21]. This suggests that *GREM1*-expressing fibroblasts are essential components of the cancer microenvironment. Additionally, we found that other skin cancers such as SCCs and MNs harbor *GREM1*-positive CAFs. It is well known that CAFs secret many soluble factors, and promote tumor growth and invasion through tumoral-stromal interactions [22, 23]. Some in vitro studies have shown that *GREM1* promotes proliferation and EMT in cancer cells [7, 14]; therefore, *GREM1* expression is likely to be one of the properties of CAFs that promote the cancer progression. However, it may be entirely possible that *GREM1* expression in CAFs is merely a marker of activated fibroblasts seen in scar tissues rather than an active promoter of cancer progression. Further investigations are required to address the exact roles of *GREM1* secreted by CAFs in the cancer microenvironment.

The strong *GREM1* expression seen in PMC stromal cells was an unexpected finding. PMCs are basaloid tumors with follicular differentiation, and are biologically benign, unless they undergo malignant transformation to pilomatrix carcinomas. A distinct histological feature exhibited by PMCs is that they often co-occur with active inflammation around tumor nodules; this inflammation arises because of foreign body reaction to keratin materials produced by the abrupt keratinization of the tumor cells. This inflammatory response may recruit the fibroblasts into the tumor, and induce *GREM1* expression in these fibroblasts. Therefore, although the tumor alone may not have invasive properties, if it could cause inflammation resulting in tissue damage and stromal reaction, this may result in recruiting the *GREM1*-expressing myofibroblasts in the tumor stroma.

Occasionally, it is challenging to differentiate BCCs from other basaloid tumors, particularly TEs. Many studies have explored the ability of immunohistochemical markers, such as BCL2, CD10, CD34, D2-40, CK15, and CK20, to help in the diagnosis of BCCs [18, 24]. We evaluated the possibility of the practical usage of stromal *GREM1* expression as a diagnostic marker; however, the low rate of stromal *GREM1* positivity (28%) in BCCs indicated that it cannot be used as a biomarker, even though all TEs were negative for *GREM1* expression. Because CD10 and CD34 are expressed by peritumoral stromal cells as well as tumor cells, we examined whether *GREM1* has any associations with expression of stromal CD10 and CD34, and found a strong correlation of *GREM1* expression with CD10 expression. However, CD10 expression did not seem to adequately co-localize with *GREM1* expression well (Fig 5). It is possible to speculate that expressions of both *GREM1* and CD10 are simultaneously induced by a factor in their microenvironment; however, they are not expressed in the same fibroblasts.

In summary, we demonstrated that the *GREM1*- positive myofibroblasts appear in scar tissue and in invasive skin cancers, including BCCs, SCCs, and MNs. Expression of *GREM1* tends to be accentuated in the tumoral-stromal interface. In BCCs, *GREM1* expression was observed mostly in the desmoplastic and mixed subtypes that induce strong stromal reactions, and appeared to be closely associated with the expression of α-SMA and CD10. Among benign skin tumors, only PMCs showed high levels of *GREM1* expression, probably due to the underlying inflammation. These findings suggest that stromal *GREM1* expression can be a marker for activated myofibroblasts in the cancer stroma or in scar tissue.

## Supporting information

S1 Fig Expression of *GREM1* in the colon mucosa analyzed by RNA in situ hybridization. (TIF)
