# Supplementary figures and images for: GREM1 is expressed in the cancer-associated myofibroblasts of basal cell carcinomas
Source: PLoS One. 2017 Mar 27;12(3):e0174565. doi: 10.1371/journal.pone.0174565 (PMC5367809; doi:10.1371/journal.pone.0174565)

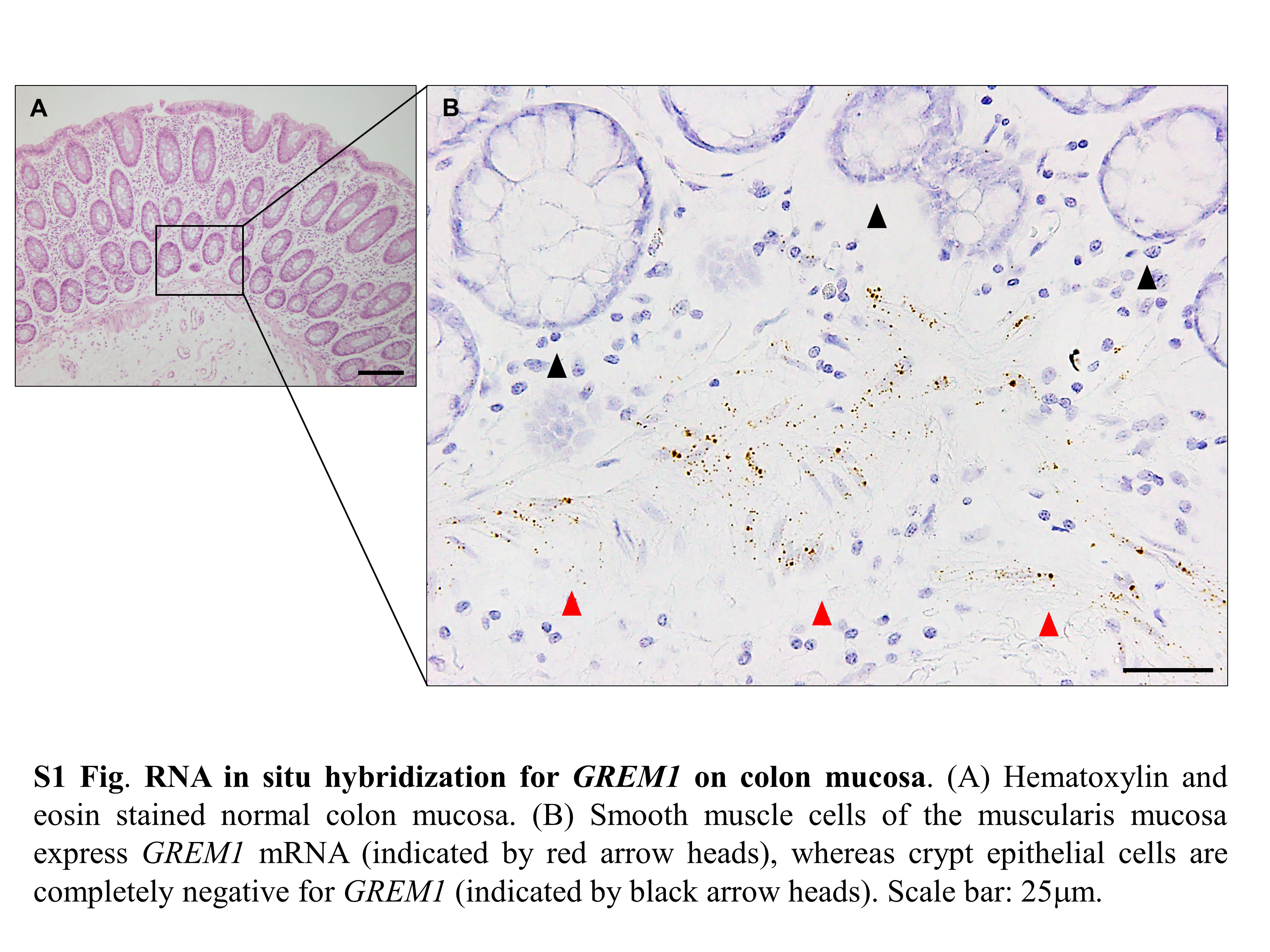

Supplement: S1 Fig — (TIF) [file pone.0174565.s001.tif]
